# Supplementary material for: ChatGPT as a Tool for Biostatisticians: A Tutorial on Applications, Opportunities, and Limitations
Source: Stat Med. 2025 Oct 23;44(23-24):e70263. doi: 10.1002/sim.70263 (PMC12548020; doi:10.1002/sim.70263)
Supplement: Supplementary file 1 — Data S1. Supporting Information. [file SIM-44-0-s001.zip › Supplementary Materials Additional.zip!/3.1_Systematic_Reviews_and_Meta_aspects/3.1_Data Extraction - o4-mini - Chat 8.pdf]

OpenAI

Use Case Meta-Analysis - Data Extraction - o4-mini - Chat 8

Rudloff et al. - 2009 - Concurrent lobular neoplasia increases the risk of ipsilateral breast cancer recurrence in situ treated with breast-conserving therapy.pdf
PDF

Vidali et al. - 2012 - Conservative treatment of breast ductal carcinoma in situ results of an Italian multi-institutional.pdf
PDF

The aim is to perform a meta-analysis on total ipsilateral local recurrence rates at 10 years in cases of ductal carcinoma in situ.

Data inclusion rules are: To ensure the validity of the follow-up data, the results should be as close to 10 years follow-up (minimum) as the information in each study allows. If there is more than one publication from an institution or group, the most recent study with appropriate follow-up will be used to extract 10-year data.

Please provide from each of the two attached studies:

- the rate of ipsilateral local recurrence for conservative surgery with additional radiotherapy (no other interventions or drugs allowed) after 10 years

- the number of eligible patients

- period of data collection

- duration of follow-up

- type of study

- age of patients (mean or median, maximum and minimum)

- treatment modalities

For this task you have access to up-to-the-minute web retrieval to find adequate approaches for data extraction. When answering the task above please proceed as follows:

1. Fetch the most relevant sources.

2. Show your step-by-step reasoning.

3. At the end, give me a concise answer citing the sources you used.

4. Please provide a dataframe with one entry for each study, and column names "study", "rate", "n_patients", "period", "follow_up", "study_type", "age", and "treatment".

Step-by-Step Reasoning

1. **Identify the Rudloff et al. (2009) study details**
    - **Period of data collection:** 1991–1995
    - **Eligible patients (surgery + RT):** 91 patients received radiation therapy (out of 294 total)
    - **10-year ipsilateral recurrence rate (surgery + RT):** 11.9%
    - **Duration of follow-up:** median 11 years (range 0–16 years)
    - **Study type:** Retrospective single-institution cohort analysis of BCT cases
    - **Patient age:** median 55 years (range 26–89 years)
    - **Treatment modalities:** Breast-conserving surgery plus external-beam tangential whole-breast RT (no chemo; 6.1% received tamoxifen, but the primary intervention was surgery + RT)
2. **Identify the Vidali et al. (2012) study details**
    - **Period of data collection:** February 1985–March 2000
    - **Eligible patients:** 586 women
    - **10-year ipsilateral recurrence rate:** 9.6%
    - **Duration of follow-up:** median 136 months (range 16–292 months)
    - **Study type:** Retrospective multi-institutional cohort study
    - **Patient age:** median 55 years (range 29–84 years)
    - **Treatment modalities:** Conservative breast surgery followed by whole-breast RT (median dose 50 Gy; boost in 50% of patients at median 10 Gy)

Concise Answer

study	rate	n_patients	period	follow_up	study_type	age	trea[illegible]
Rudloff et al. 2009	11.9%	91	1991–1995	median 11 yr (0–16 yr)	Retrospective single-institution cohort	median 55 yr (26–89 yr)	Breast-cons[illegible] surgery + e[illegible] whole-brea[illegible] chemo; 6.1[illegible]
Vidali et al. 2012	9.6%	586	Feb 1985–Mar 2000	median 136 mo (16–292 mo)	Retrospective multi-institutional cohort	median 55 yr (29–84 yr)	Conservati[illegible] surgery + w[illegible] RT (median[illegible] in 50% at m[illegible]

References

- Rudloff U, et al. Concurrent lobular neoplasia increases the risk of ipsilateral breast cancer recurrence in DCIS treated with breast-conserving therapy. Cancer. 2009;115:1203–14.
- Vidali C, et al. Conservative treatment of breast ductal carcinoma in situ: results of an Italian multi-institutional retrospective study. Radiat Oncol. 2012;7:177.

Quellen
